# Surface Proteoglycans as Mediators in Bacterial Pathogens Infections

**DOI:** 10.3389/fmicb.2016.00220

**Published:** 2016-02-24

**Authors:** Beatriz García, Jesús Merayo-Lloves, Carla Martin, Ignacio Alcalde, Luis M. Quirós, Fernando Vazquez

**Affiliations:** ^1^Department of Functional Biology, Microbiology, Faculty of Medicine, University of OviedoOviedo, Spain; ^2^Instituto Oftalmológico Fernández-Vega, Fundación de Investigación Oftalmológica, Universidad de OviedoOviedo, Spain; ^3^Department of Surgery, University of OviedoOviedo, Spain; ^4^Service of Microbiology, Central University Hospital of AsturiasOviedo, Spain

**Keywords:** infection, bacteria, host interaction, proteoglycans, glycosaminoglycans

## Abstract

Infectious diseases remain an important global health problem. The interaction of a wide range of pathogen bacteria with host cells from many different tissues is frequently mediated by proteoglycans. These compounds are ubiquitous complex molecules which are not only involved in adherence and colonization, but can also participate in other steps of pathogenesis. To overcome the problem of microbial resistance to antibiotics new therapeutic agents could be developed based on the characteristics of the interaction of pathogens with proteoglycans.

## Introduction

According to the World Health Organization (WHO), in high-income countries people predominantly die of chronic diseases, whereas infectious diseases in low-income countries cause almost one third of all deaths (World Health Organization, 2014b). It is necessary to focus research not only on currently non-controlled infectious pathologies, but also on those pathogens which have hitherto been considered controlled in high income countries but are now re-emerging, as well as newly emerging diseases. One of the most important reasons behind the emergence or re-emergence of a disease is the fact that most such pathogens are able to evolve quickly, thus acquiring transmissible genetic modifications that give them an advantage in overcoming both the host and environmental changes, as in the case of the evolution and the transference of antibiotic resistance genes in current generation of drug-resistant strains of bacteria. The spread of these resistant strains is favored by both global trade and the mobility of individuals, resulting in infectious diseases becoming a global health problem (Morens and Fauci, 2013). The report “Antimicrobial resistance: global report on surveillance,” published by the WHO in April 2014 and based on data from 114 countries worldwide, highlights the problem of the large increase in antimicrobial resistance in all regions of the world (World Health Organization, 2014a). Among other things, the WHO has drawn attention to the need to develop new diagnostic products, antibiotics and other instruments that allow health professional to combat emerging resistances.

Bacterial adherence and colonization of host cells are the crucial initial steps for infection to occur. Innovative strategies of interference in bacterial adhesion need to be considered and developed by investigating the mechanisms used by bacteria to overcome host defenses and attach to host cells. Bacteria possess diverse adherence mechanisms, including pili, fimbriae and various types of membrane proteins, all of which can exist in the same pathogen and co-operate to increase bacterial adhesion (Virji, 2009).

**FIGURE 1:**
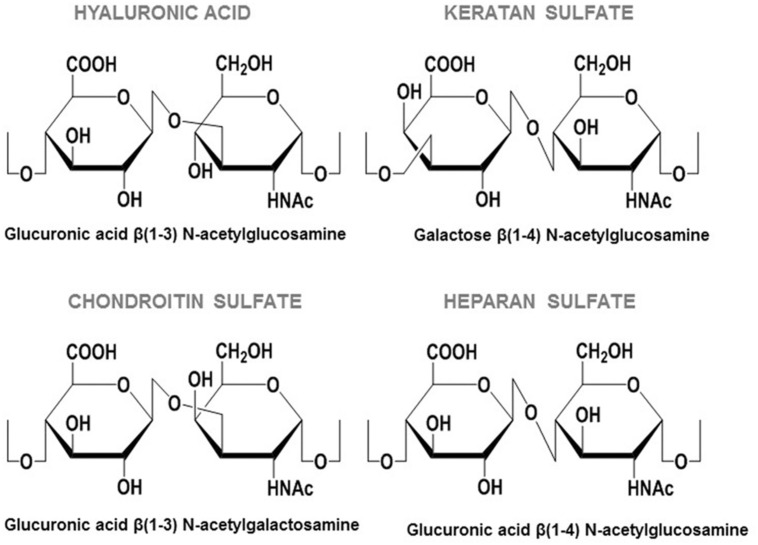
**Repeating disaccharide units of GAGs**.

**FIGURE 2:**
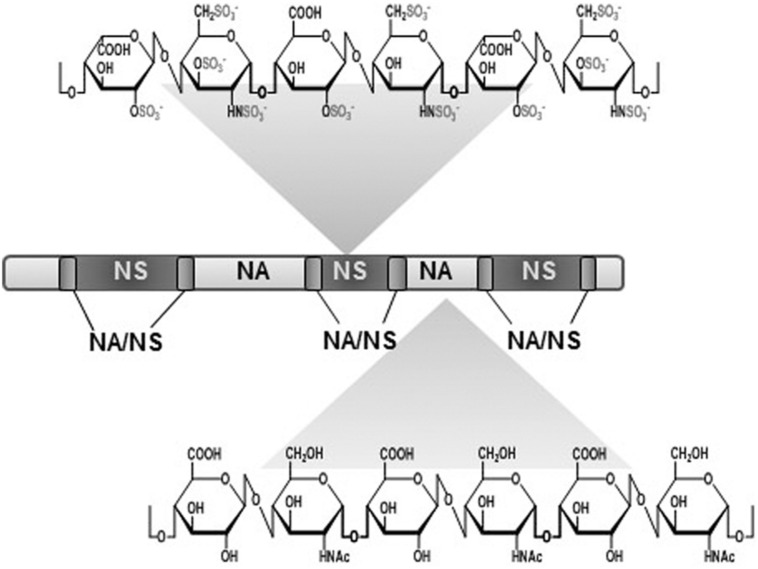
**Heparan sulfate (HS) domain architecture.** The highly sulfated NS-domains are flanked on either side by less-sulfated transition zones (NA/NS). In turn, these sulphated regions are separated by non-sulfated NA-domains.

An interesting feature of the molecules used as receptors by pathogens is their wide distribution in tissues, while simultaneously presenting a certain degree of variability that enables the tropism known to exist in some bacterial adhesions. Proteoglycans (PGs) meet both these conditions; they are complex ubiquitous molecules which have a different distribution and composition depending on the tissue (Esko et al., 2009). PGs consist of different types of core protein modified with chains of anionic polysaccharides called glycosaminoglycans (GAGs). GAGs are mainly made up of repeated disaccharide units, and depending on the composition of these units they can be classified as either: heparin/heparan sulfate (HP/HS), chondroitin/dermatan sulfate (CS/DS), keratan sulfate, and hyaluronic acid (HA; Esko et al., 2009; **Figure 1**). HS, composed of glucuronic acid and *N*-acetyl glucosamine, is the most widespread and physiologically relevant GAG (Sugahara and Kitagawa, 2002). It has a complex biosynthesis: after the polymerization of the sugar backbone, the chains are modified in different interdependent reactions, including *N*-deacetylation/*N*-sulfatation, epimerization, and various *O*-sulfations. This modification process is carried out by a number of correlated enzymes which act in a certain order. The first reaction, *N*-deacetylation/*N*-sulfatation, generates a domain organization of HS chains that includes *N*-acetylated (NA) and *N*-sulfated (NS) domains, which are separated by mixed NA/NS domains. Most subsequent modifications take place in NS domains, which become highly modified and hypervariable (Sugahara and Kitagawa, 2002). It has been described that NA/NS domain length and NS domain sulfation patterns are characteristic of the type of cell involved, and on its physiological state (Sugahara and Kitagawa, 2002; **Figure 2**).

**FIGURE 3:**
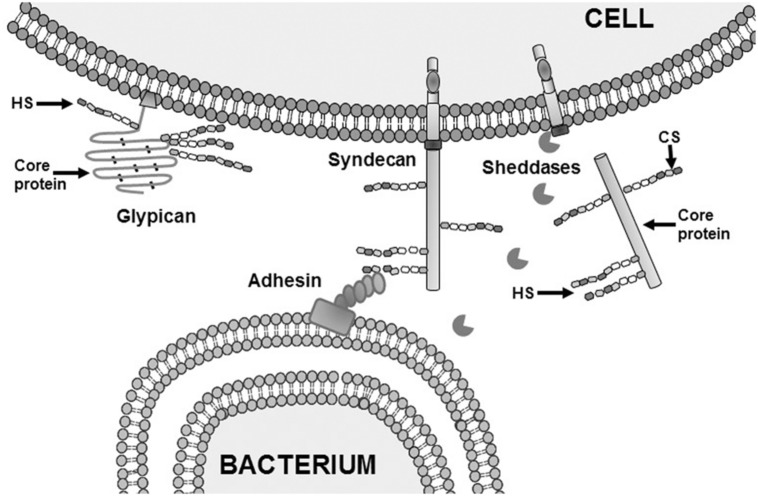
**Adhesion of bacterium to target cells mediated by HS and shedding of syndecan ectodomains.** Schematic representation of the interaction between an adhesin on the outer membrane of a Gram negative bacteria and HS from syndecan on the cell surface of a eukaryotic.

Proteoglycans with HS moieties (HSPGs) can be classified according to their location: on the cell surface, where two families are found: the transmembrane syndecans and the glycosyl phosphatidylinositol-anchored glypicans; in the extracellular matrix (ECM) where there are three types of HSPGs: agrin, perlecan and type XVIII collagen; and inside intracellular vesicles, where serglycin is located (Sarrazin et al., 2011; **Table 1**). HSPGs have multiple functions, some of them dependant on the core proteins, but most related with the GAG chains because of their characteristic epimerization and sulfation pattern. This structural diversity allows HSPGs to play a key role in many processes including cell adhesion and migration, organization of ECM, regulation of proliferation, differentiation and morphogenesis, cytoskeleton organization, tissue repair and inflammation. HSPGs can bind several ligands such as cytokines, chemokines, growth factors, and morphogens, protecting them against proteolysis and controlling their gradients (Sarrazin et al., 2011). HSPGs co-operate with different molecules to define basement membrane structure and to mediate in cell-ECM attachment, cell–cell interactions, and cell motility (Sarrazin et al., 2011). Shedding of membrane-bound HSPG ectodomains can be carried out by enzymatic cleavage (Garton et al., 2006; Nam and Park, 2012; **Figure 3**), and is an important factor in host response to tissue injury and inflammation in pathophysiological processes (Garton et al., 2006).

**Table 1:** Classification of HSPGs according to their location.

Heparan sulfate proteoglycans	
Cell surface	Transmembrane syndecans
	Glycosylphosphatidylinositol-anchored glypicans
Extracelullar matrix	Agrin
	Perlecan
	Type XVIII collagen
Intracellular vesicles	Serglycin


HS moieties are involved in the adherence of many microorganisms. This ranges from attaching normal human microbiota to cell surfaces and includes the genus *Lactobacillus*, which interacts primarily with HS and CS in the vaginal and intestinal epithelium (Martín et al., 2013), to various pathogenic bacteria, viruses, and parasites. Pathogens interact with HPSGs not only to achieve adherence and colonization, but also in invasion, internalization, dissemination, and toxicity (Kamhi et al., 2013; García et al., 2014). Some bacterial pathogens are even able to induce shedding of cell surface HSPGs, particularly syndecan-1. The released syndecan-1 ectodomain then binds to host defense factors, such as antimicrobial peptides and cytokines, which then become inhibited in a HS-dependent manner, resulting in deregulation of the infection response and the promotion of pathogenesis (Menozzi et al., 2002; Barlett and Park, 2010).

This review focuses on describing the role of PGs and GAGs in the principal human infectious diseases caused by bacteria (**Table 2**).

**Table 2:** Diverse infectious pathologies caused by bacteria and mediated by GAGs, indicating the molecular species involved.

Localization	Pathology	Bacteria	GAGs
Respiratory tract	Pneumonia	*Streptococcus pneumoniae*	HS, CS
	Pneumonia	*Haemophilus influenzae*	HS
	Pneumonia	*Chlamydia pneumoniae*	HS
	Tuberculosis	*Mycobacterium tuberculosis*	HS
	Cystic fibrosis	*Pseudomonas aeruginosa*	HS
	Pertussis	*Bordetella pertussis*	GAGs
	Pharyngitis	*Streptococcus pyogenes*	HS, DS
Systemic	Lyme disease	*Borrelia burgdorferi*	HS, DS
Central nervous system	Meningitis	*Streptococcus agalactiae*	GAGs
	Meningitis	*Streptococcus pneumoniae*	HS, CS
	Meningitis	*Neisseria meningitidis*	HS, CS
Gastrointestinal tract	Gastritis, ulcers	*Helicobacter pylori*	HS
	Inflammation	*Enterococcus faecalis*	HS
	Inflammation	*Streptococcus agalactiae*	HS
	Inflammation	*Staphylococcus aureus*	HS
	Listeriosis	*Listeria monocytogenes*	HS
Urogenital tract	Gonorrhea	*Neisseria gonorrhoeae*	HS
	Urogenital chlamidiasis	*Chlamydia trachomatis*	HS, DS
Cornea	Keratitis	*Staphylococcus aureus*	HS
	Keratitis, ulcers	*Pseudomonas aeruginosa*	GAGs


## Infectious Respiratory Diseases

Common upper respiratory infections include the common cold, tonsillitis, pharyngitis, epiglottitis, and laryngotracheitis. Infections of the lower respiratory tract include bronchitis, bronchiolitis, tuberculosis, and pneumonia. Flu may affect either the upper or lower respiratory tract (Dasaraju and Liu, 1996). The WHO states that the leading infectious cause of death is lower respiratory infections, which caused more than 3 million deaths worldwide in 2012 (World Health Organization, 2014b).

Pneumonia, one of the most common lower respiratory infections, is an inflammation of the lung parenchyma, commonly caused by viruses or bacteria when the functioning of the host’s immune system is reduced.

The most frequent type of pneumonia is caused by streptococci, particularly by *Streptococcus pneumoniae*. This bacterium is also a significant cause of meningitis, bacteremia, and otitis media (Tuomanen et al., 1987; Hollingshead and Briles, 2001). During the colonization step*, S. pneumoniae* binds to mucosal epithelial cells using HS and CS as receptors (Tonnaer et al., 2006). Additionally, the pathogen produces extracellular glycosidases which modify various glycans and GAGs in the human airway epithelium and, as a consequence, more receptors for adherence are revealed, and the sugars released are used by the pneumococci for growth. The glycosidases secreted also negatively affect other bacteria, giving the pathogen an advantage in interspecies competition (King, 2010). Furthermore, *S. pneumoniae* has the ability to induce the ectodomain shedding of syndecan-1 from the cell surface. In fact the TIGR4 strain directly stimulates shedding through a ZmpC metalloproteinase in an intracellular signaling-independent manner in order to promote its pathogenesis. However, other strains do not express ZmpC and they shed syndcan-1 through an as yet unknown alternative mechanism (Chen et al., 2007).

*Haemophilus influenzae* constitutes the second most common cause of bacterial pneumonia. Strains of this bacterium are classified into typeable and non-typeable (NTHi), depending on the presence or absence, respectively, of a polysaccharide capsule (King, 2012). NTHi strains are the main cause of infections in the respiratory tract, affecting mainly non-ciliated cells or damaged mucosa (King, 2012). NTHi has several different mechanisms for adherence to mucosal surfaces, e.g., five types of pili and a large number of surface proteins, including diverse adhesins such as protein E, Hia, Hap, and protein F (Moulder, 1991). Approximately 75% of NTHi strains express the adhesins HMW1 and 2 (high molecular weight proteins 1 and 2), which bind to sulfated GAGs, particularly to HS chains (Finney et al., 2014). NTHi can undergo phase variation to promote the persistence of bacteria (Noel et al., 1994).

Another causative agent of bacterial pneumonia is the obligate intracellular human pathogen *Chlamydia pneumoniae*. This microorganism has a unique biphasic life cycle with two forms, the elementary bodies (EBs) and reticulate bodies (RBs). EBs are responsible for infecting host cells and promoting entry, after which they transform into RBs. This intracellular RB form uses the host metabolism to replicate and reorganize itself back into EBs, which are then released into the lung by cell lysis and enable the infection of new cells (Moulder, 1991). The initial attachment of EBs to the epithelium surface is mediated by HS chains, mainly through electrostatic interactions, although other co-receptors may be required for efficient attachment and entry (García et al., 2014).

Another important infection of the lower respiratory tract is tuberculosis. According to the WHO, this disease is the second leading cause of death attributable to a single infectious agent (World Health Organization, 2015). The infection commonly begins in the lungs but can spread to other tissues through the expression of a heparin-binding protein (Chen et al., 2008). *Mycobacterium tuberculosis* adheres to pulmonary epithelial cells, although the microorganism is also able to infect phagocytes. The bacteria’s adherence to the epithelium is mediated predominantly by heparin-binding hemagglutinin adhesin (HBHA), which interacts with HS chains. HBHA also mediates in the triggering mechanisms for the transcytosis process, which leads to extrapulmonary dissemination of the infection (Menozzi et al., 2006; García et al., 2014).

The microorganism *Pseudomonas aeruginosa* is an opportunistic pathogen that is, among other diverse infections, the major cause of burn infections and cystic fibrosis (CF). This bacterium uses diverse strategies, which may act independently or in combination, including type IV pili and adhesins, to produce a wide range of infections (Barlett and Park, 2011). The pathogen needs an injured respiratory epithelium in order to bind properly as such damage affects the tight junctions between epithelial cells and leaves the basolateral receptors exposed (Barlett and Park, 2011). In polarized cells, *P. aeruginosa* uses different receptors depending on the side of the cell involved, binding to complex *N*-glycans on the apical surface and to HS moieties of HSPGs on the basolateral surface (Barlett and Park, 2011). During injury and dedifferentiation of epithelium, cells are not completely polarized, and levels of HSPGs are increased on the apical surface. In these circumstances, both HSPGs and *N*-glycans act as receptors for the pathogen on the apical surface. Another strategy employed by *P. aeruginosa* to increase its pathogenicity is the virulence factor LasA, a zync metalloendopeptidase, which is able to induce syndecan-1 shedding indirectly (Barlett and Park, 2011). This shedding downregulates the host defenses, leading to increased bacterial virulence and enhances its survival (Chen et al., 2008). Circulating GAG levels in CF patients are increased, not only due to shedding, but also because of other bacterial exoenzymes which are produced to inactivate the action of molecules from the host immune system. *P. aeruginosa* produces proteinases, elastase, and alkaline proteinase all of which release DS from matrix PG decorin, which then binds to neutrophil-derived α-defensin, whose bactericidal activity is thereby neutralized (Schmidtchen et al., 2003; Barlett and Park, 2010). Released GAGs are also able to interact with LL-37 electrostatically, inhibiting its binding with bacteria and in this way disabling its bactericidal action. Unconjugated LL-37 peptide can be degradated through proteolysis by neutrophil elastase and cathepsin D, which is induced by *P. aeruginosa* (Kamhi et al., 2013).

Pertussis is a highly contagious bacterial disease of the respiratory tract that mainly affects infants and young children. The WHO estimates that about 16 million cases of pertussis occurred worldwide in 2008, and it continues to be a public health concern even in countries with high vaccination coverage (World Health Organization, 2011). The infection is caused by *Bordetella pertussis*, which expresses filamentous hemagglutinin adhesin (FHA), which binds to sulfated GAGs by its C-terminal to initiate the infection of bronchial epithelial cells (Hannah et al., 1994).

The pathogen *Streptococcus pyogenes* is a member of Group A streptococci. This microorganism is able to infect different human tissues, including the respiratory tract. The bacterium interacts with different GAGs from cells surfaces, mainly with DS and HS, through different types of protein M, its major surface-expressed virulence factor (Frick et al., 2003).

## Systemic Infection Diseases

In some infections, the bacterial pathogens are distributed through multiple tissues. An example is Lyme disease, a chronic multisystem disorder caused by the obligate intracellular pathogen *Borrelia burgdorferi*, in which the skin, heart, joint, skin, and nervous system may be affected This bacterium has multiple surface proteins with different binding specificities to GAGs depending on the tissue affected (Leong et al., 1998). In addition, different GAGs act as receptors for *B. burgdorferi* depending on the host cells; both HP and HS are essential in adherence to primary endothelium and adult kidney Vero cells, but only DS is involved in attachment to human embryonic kidney cells, while all these GAG species mediate adherence to HeLa cells, neuronal and primary telencephalon cell lines (Leong et al., 1998; García et al., 2014).

Sepsis is the systemic host response to microbial infections, and, among other alterations, this involves an increase in the permeability of the endothelium caused by the shedding of its PGs (Henrich et al., 2010). Shedding of syndecan-1 is stimulated by proinflammatory substances and, as a consequence, levels of circulating syndecan-1 increase, thereby promoting leukocyte adherence (Nelson et al., 2008). This increase in syndecan-1 levels is correlated with the cardiovascular SOFA (Sequential Organ Failure Assessment) score. During septic shock there are high levels of some GAGs in plasma and, although there is no correlation with syndecan-1 levels, they are correlated with mortality (García et al., 2014; Nelson et al., 2014). Specifically, the quantity of circulating HS and HA increases, while KS levels are moderately reduced, and those of CS remain unaltered (Nelson et al., 2014). These released GAGs are mainly from the altered endothelium, but they can also come from connective tissue and basement membrane (Nelson et al., 2014).

## Nervous System Infections

Certain microbes invade brain endothelial cells and breach the blood–brain barrier (BBB) through interactions with GAGs, thus establishing central nervous system infections (Chang et al., 2011).

Meningitis has been declared one of the top 10 causes of mortal infection worldwide, and is particularly devastating in newborns (World Health Organization, 2014b). The most common agent of neonatal bacterial meningitis is *Streptococcus agalactiae*, which uses alpha C protein (ACP) to interact electrostatically with GAGs and cross the BBB. ACP also mediates streptococci entry into epithelial cells, involving Rho GTPase-mediated actin polymerization (García et al., 2014). GAG expression patterns are important for adherence in different cells, since they determine the efficiency of bacterial dissemination during infection by *S. agalactiae* (Chang et al., 2011).

In older children and adults, meningitis is mainly caused by *S. pneumoniae* and *Neisseria meningitidis*. As described above, the binding of *S. pneumoniae* to cells happens through interactions with sulfated GAGs, mainly HS and CS4 (Tonnaer et al., 2006). The pathogen *N. meningitidis* has a large number of molecules involved in adhesion including adhesin OpC, which binds to HS to initiate epithelial cell invasion (de Vries et al., 1998).

## Gastrointestinal Infection Diseases

More than 1200 bacterial species can inhabit the healthy human gastrointestinal tract, and there is not yet general agreement on a reliable total number of species (Rajilić-Stojanović et al., 2007).

The pathogenic bacterium *Helicobacter pylori* appears adhered to human gastric mucosa, and its presence is associated with chronic gastritis type B and ulcers. What is more, *H. pylori* has been found to be related with gastric cancer, and has been designated a class I carcinogenic agent by the International Agency for Research on Cancer (García et al., 2014). Different outer-membrane proteins of *H. pylori* bind to HS from gastric cells to bring about adhesion (Guzman-Murillo et al., 2001). The pathogen also secretes various proteins that also interact with HS chains, such as cytotoxin vacA. This toxin attaches and enters into cells inducing vacuolation, which in turn leads to host cell death (Palframan et al., 2012). The most pathogenic strains of *H. pylori* have the cag pathogenicity island, which encodes the cytotoxic protein CagA, and a type IV secretion system that injects this protein into the gastric cells (Odenbreit et al., 2000). Protein CagA affects intracellular signaling pathways, including the NF-kB pathway, which leads to upregulation of the expression of the gene encoding syndecan-4, which is commonly found overexpressed in carcinoma cell lines (Odenbreit et al., 2000).

Some of the species that reside in the human intestinal tract are typically associated with systemic infection in post-surgical, shock, and in trauma patients. This is the case of *Enterococcus faecalis*, the major cause of nosocomial infections, which affects a variety of tissues (Baldassarri et al., 2005). Enterococci use HS for initial adherence and for their internalization in professional and non-professional phagocytes, which is mediated by triggering cascades of protein kinases and the reorganization of microtubules (Kamhi et al., 2013). *E. faecalis* is able to resist specific killing mechanisms inside these phagocytes and thus continue its invasion and dissemination (Baldassarri et al., 2005). Although syndecan-1 is widely expressed in enterocytes, it has been proven not to be essentially involved in the adhesion of any enterococci (Sava et al., 2009). However, it does play a role in interactions with other enteropathogenic bacteria, such as *Listeria monocytogenes*, staphylococci and streptococci, which may use the intestinal tract as a portal of entry in special circumstances (Sava et al., 2009).

Listeriosis is a serious infection caused by eating food contaminated with the intracellular pathogen *L. monocytogenes*. The bacterium employs different membrane proteins to adhere to epithelial cells using HSPGs, particularly syndecan-1 (Henry-Stanley et al., 2003). *L. monocytogenes* has great invasive capacity, thanks to its ability to induce its own internalization in a wide range of cells (Henry-Stanley et al., 2005). In phagocytic cells, the bacterial surface protein ActA plays two important roles: extracellularly, it mediates adhesion by binding with low affinity to HSPGs; intracellularly, it directs actin assembly which is needed for the bacterium’s intracellular life-cycle and motility (García et al., 2014). In non-phagocytic cells, the internalization of *L. monocytogenes* is mediated by one of two invasins, depending on the polarization of the cell. In polarized epithelial cells, invasin InlA uses molecule E-cadherin in junctional sites. However, for internalization in non-polarized cells, *L. monocytogenes* utilizes InlB to interact with GAGs through the hepatocyte growth factor receptor Met; the formation of this complex promotes the endocytosis of the receptor with the bound bacterium (Pizarro-Cerdá and Cossart, 2006).

Although *S. aureus* is a member of the normal human microbiota, it is a common cause of infections affecting different parts of the body (Barlett and Park, 2010). The adherence of the bacterium to the intestinal epithelium and its internalization involve various mechanisms. One is mediated by two bacterial fibronectin binding proteins (FnBPs) that interact with the β1 integrin (Hauck and Ohlsen, 2006; Hess et al., 2006). An alternative mechanism involves the interaction between a heparin-binding protein and HSPGs, mainly syndecan-1 (García et al., 2014), which allows adherence and internalization via non-professional phagocytes in a fibronectin independent way (Hess et al., 2006).

A group B streptococci, *S. agalactiae*, occasionally colonizes mucosal surfaces of the human gastrointestinal tract. This pathogen is potentially highly invasive and dangerous in infants, in the elderly and in diabetic patients. As in cases of its adhesion and crossing of the BBB, the bacterium uses ACP to interact with HSPGs, mainly syndecan-1, for adherence and entrance into cells, through an actin-dependent mechanism (Kamhi et al., 2013).

## Sexual Infectious Diseases

Sexually transmitted infections can be caused by more than 30 different bacteria, viruses, and parasites (World Health Organization, 2013). One of the most important sexually transmitted diseases is gonorrhea, caused by *Neisseria gonorrhoeae*. The bacterium employs diverse strategies for adherence to cell surfaces, including pili and several Opa adhesins, such as OpaA, which uses syndecan-1 and -4 as receptors (García et al., 2014). Following the interaction with OpaA, the cytoplasmic domains of syndecans trigger intracellular signaling cascades, activating phosphatidylcholine-specific phospholipase C and the acid sphingomyelinase, which leads to ceramide formation. Ceramide mediates in the reorganization of actin and endocytosis of gonococci. The syndecan-Opa complexes can also induce protein kinase C activation via an integrin-mediated mechanism, through binding to the serum ECM proteins fibronectin and vitronectin (Kühlewein et al., 2006).

Another common pathogen in sexual disseases is *Chlamydia trachomatis*. The bacterium has diverse serovars, which cause different diseases: serovars A-C cause trachoma; serovars D-K are involved in sexually transmitted infections and serovars L1, L2, and L3 cause lymphogranuloma venereum. The individual serovars have different strategies for adhesion and colonization (Becker, 1996). *C. trachomatis* is able to use diverse binding mechanisms, including some that involve direct interaction with HS on cells. Depending on the serovar, the degree of HS involvement varies, it being essential for serovar L2 binding but not for serovar E (Taraktchoglou et al., 2001). Additionally, *C. trachomatis* is able to adhere to and enter cells by indirect ways due to the interaction between its EB infectious forms and fibroblast growth factor 2 (FGF2; Kim et al., 2012). The complex thus formed interacts with the FGF2 receptor, which is locally activated and mediates in EB internalization into cells. The EB-FGF2 complex may involve synergistic interactions with the EB membrane bacterial protein OmcB, which also interacts with HS from the cell surface (Fechtner et al., 2013). The internalization of bacteria increases the production of FGF2, triggering a positive feedback which upregulates the infection. FGF2 may also play additional roles in enhancing pathogenesis by potentiation of inflammatory response, inhibition of apoptosis and regulation of gene expression (Kim et al., 2012).

## Ocular Infectious Diseases

There is a wide range of non-specific pathogens which are able to infect certain areas of the eye, mainly conjunctiva, lid, and cornea. The most common external ocular infections are caused by bacteria, which mainly interact with HS chains as receptors (Armstrong, 2000). Most keratitis are caused by *S. aureus* and *P. aeruginosa.* As described above, both interact with HSPGs from injured epithelium, a requirement for corneal invasion.

The adherence of *S. aureus* to corneal epithelium takes place via collagen-binding adhesin and FnBPs A and B, which interact with collagen and fibronectin, respectively (Armstrong, 2000). Syndecan-1 is not involved in the initial attachment, although *S. aureus* induces its shedding via α- and β-toxin (Barlett and Park, 2010; Hayashida et al., 2011). Syndecan-1 ectodomains increase pathogenicity and the potential for bacterial survival by interacting with neutrophils, whose antibacterial function is thus inhibited. The shedding of syndecan-1 also affects the inflammatory process by generating gradients of quimioatractors (Hayashida et al., 2011).

Severe keratitis and central corneal ulcers are caused by *P. aeruginosa* infections (Fleiszig and Evans, 2002). The initial attachment is mediated by surface structures such as pili and polysaccharides, which mediate biofilm formation over the epithelium and on contact lenses. Extended contact lens wearing increases bacterial adherence to corneal epithelium (Fleiszig and Evans, 2002). The bacterium is not only able to adhere to cell surfaces, but it also interacts with perlecan from ECM (Willcox, 2007). As was described earlier, the bacterium is able to produce several exoproducts that degrade PGs and produce tissue injury by degradation of basement membranes and the ECM, promoting bacterial virulence and invasion both individually and via quorum-sensing (Twining et al., 1993; O’Callaghan et al., 1996; Chen and Hazlett, 2001). The bacterium *P. aeruginosa* also induces the production of host exoproducts that contribute to corneal damage and to an excessive activation of the host defense system (Twining et al., 1993).

## Glycosaminoglycans as Therapeutic Targets

The increase in the emergence of antibiotic resistant bacterial pathogens has limited the efficacy of existing treatments in infections. New therapies which can supplement or replace old treatments avoiding the selective pressures that lead to bacterial resistance should be investigated and developed urgently. GAGs have been widely explored and used as therapeutic agents in diverse biomedical treatments. This broad application of GAGs is due to their involvement in multiple physiological processes such as coagulation, thrombosis, inflammation, cancer, angiogenesis, cell differentiation, tissue repair, and microbial infections (San Antonio and Iozzo, 2001).

The therapeutic applications of certain GAGs are well-known, including the use of HA in various joint disorders and plastic surgery (Kogan et al., 2007), of HP as an anticoagulant and anti-inflammatory agent (Young, 2008), and of CS in the treatment of osteoarthritis (Clegg et al., 2006). In the last few years, GAG molecules have begun to be used as drug delivery agents in nanomedicine. GAGs interact electrostatically with different compounds or biologics used for therapeutic purposes thanks to their strong negative charge. The drugs are delivered systemically or locally to treat a variety of pathological conditions, including cancer, glaucoma, wounds, and burns (Misra et al., 2015).

The increased understanding of the structure–function relationship of GAGs has made possible the design of new compounds with a potential therapeutic role in a variety of diseases (Raman et al., 2005; Gandhi and Mancera, 2008). Some analogs and antagonists of these molecules, with suitable charge and conformation, have already been developed and used to affect some GAG functions, including anticoagulants, anti-inflammatories, antimetastatics, and also to interfere with their ability to interact with growth factors, proteases, and different host defense molecules, such as neutrophils, proinflammatory molecules, and antimicrobial peptides (Rusnati et al., 2005; Rusnati and Urbinati, 2009; Harris et al., 2014). The use of GAG based-molecules in the field of cancer is particularly interesting, several compounds having already entered the clinic, and one of them (PI-88) currently being in phase III trials (Ferro et al., 2007). Since different GAGs mediate interactions with pathogens, the characterization of the different roles played by these molecules in several infectious pathologies is essential in order to design new therapeutic molecules based on these polysaccharides which may interfere in the establishment of microorganisms.

A layer of extracellular GAGs covers the healthy urothelium; these molecules attract water, creating a chemical barrier that protects against infections. During urinary tract infections the layer of GAGs becomes damaged. The restoration of this layer with GAGs supplied exogenously has been shown to play a protective role against recurrent infections and inflammatory factors (Yazawa, 2014). Other experiments have demonstrated that intravesical therapy using some GAGs, specifically HA and CS, was more effective than antibiotic therapy in reducing recurrent episodes of infections in the urinary tract (Torella et al., 2013). Natural or synthetic GAGs can be used as prophylactic agents in treatments for recurrent urinary tract infections, and even as adjunctive therapy with classic anti-infective treatments.

Hyaluronic acid has also been useful in different therapeutic aplications, such as in the treatment of chronic bronchitis. The treatment of such patients with HA led to a reduced number of acute episodes than in patients treated with a placebo. These experiments suggest that HA reduces aggravation in patients with chronic bronchitis and may help to decrease the use of antibiotics in their therapies, diminishing the risk of generating antibiotic resistance. The investigators involved in this trial proposed that HA possibly enhances host cellular defense mechanisms (Venge et al., 1996; Souza-Fernandes et al., 2006).

Therapeutic GAG-like molecules can come from different natural sources, including mammalian tissues, non-mammalian origins such as invertebrates, and synthetic GAG mimetics. These molecules can act both as agonist or antagonist either by interfering with endogenous GAGs or by forming complexes with protein ligand and/or receptors (Rudd et al., 2012; Pomin, 2015). The possible role of GAG analogs in inhibiting pathogen interaction has been widely studied, especially in virus attachment and dissemination.

Heparin derivatives and heparin-mimicking molecules are called heparinoids, and they have been used in many therapeutic applications as inhibitors of HS-protein interactions. Fucoidans are heparinoids obtained from marine brown algae. For the past decade fucoidans isolated from different species have been extensively studied due to their wide variety of biological activities, but their potent anticoagulant action is by far the most widely investigated. These molecules also have different anti-infective activities, including antiviral activity against a wide range of viruses via receptor entry blocking or interference with replicative processes (Li et al., 2008). Various experiments have demonstrated that fucoidans also have antiparasitic activities against *Toxoplasma gondii*, *Plasmodium*, and *Leishmania*. Moreover, recent *in vitro* and *in vivo* studies have related treatment with fucoidans with prevention of infection by *H. pylori* through the inhibition of adhesion to mucosal surfaces (Fitton, 2011). All these results show the potential of these molecules to be used as prophylaxis measures.

Another heparinoid analog of HS is suramin, which also presents different therapeutic activities, mainly antiangiogenic, antiviral – through its inhibition of the reverse transcriptase of several retroviruses (Wang et al., 2014), and antiparasite (McGeary et al., 2008). The effect of suramin against pathogenic bacteria has also been analyzed in different studies and it has been found to be able to produce non-specific antibacterial resistance against *Mycobacterium bovis* and *L. monocytogenes* through macrophage activation*s* (Brandely et al., 1986). This analog is also a potent inhibitor of RecA protein of *M. tuberculosis*, the protein that is responsible for the development of antibiotic resistance (Nautiyal et al., 2014).

Heparosan, produced and found in the capsule of some pathogenic bacteria such as *Escherichia coli* K5 and *Pasteurella multocida*, is a molecule whose structure is similar to unmodified natural HS. Heparosan can be modified by chemical or enzymatic methods, thereby creating new derivatives capable of affecting different processes, such as molecules that display antiviral activity *in vitro*, especially against the human immunodeficiency virus (HIV), herpes simplex virus, and human papilloma virus (Li et al., 2013). Both HP and heparosan are able to block the adhesion of pathogenic *E. coli, Pasteurella multocida, S. aureus* to enterocytes, while *Lactobacillus rhamnosus* adherence is not altered. In addition, treatment with probiotics supplemented with heparosan has been suggested as a preventive treatment for infections (Chen et al., 2012).

Chondroitin derivatives have also been found in nature, such as fucosylated CS (FucCS) from sea cucumber species. This molecule possesses diverse activities, such as anti-coagulant, anti-thrombotic, anti-inflammatory, and anti-cancer actions. FucCS is also able to block HIV virus entry into cells (Huang et al., 2013) and *P. falciparum* cytoadherence to endothelial cells (Bastos et al., 2014).

Other GAG analogs displaying a suitable charge and conformation can interfere with the electrostatic interaction of the GAGs themselves with host defense molecules. The bactericidal activity of some cationic antimicrobial peptides, such as LL-37, is disabled when the peptides bind to GAGs. The electrostatic interaction of LL-37 with these molecules can be disrupted by nebulized hypertonic saline treatment, included in the therapy against airway mucus of CF patients. The release of LL-37 results in a restoration of the peptide antimicrobial activity (Reeves et al., 2011).

In addition to molecules of various natural origins, knowledge of the relation between the structure and function of GAGs has allowed the design and development of various families of synthetic derivatives for different therapeutic purposes. Semi-synthetic glycosaminoglycan ethers (SAGEs) are derived from sulfation and alkylation of HA; different SAGEs can be used in the treatment and prevention of different pathologies, including cancer (Prestwich and Kennedy, 2011), or different ophthalmic therapies (Prestwich et al., 2012). SAGEs can also play a role in infection treatments; gingivitis and periodontitis are initiated by chronic bacterial infections caused among others by *Porphryomonas gingivalis*. This therapy can be a major advantage compared to treatment with antibiotics which may lead to the generation of resistant bacteria.

ReGeneraTing Agents (RGTAs) are synthetic HS mimics resistant to digestion with multiple endoglycanases such as heparanase, chondroitinase, hyaluronidase, and dextranase. They are able to interfere in the interaction of GAGs with different ligands, including various growth factors such as FGF1, FGF2, and VEGF, and can play an important role in wound regeneration. CACIPLIQ20^®^ (OTR3, Paris, France) is an RGTA member specifically designed to restore the damaged ECM in chronic wounds. Indirectly, these molecules, based on GAGs, may be useful in preventing infections in open wounds and thus reducing the time of exposure to pathogens (Barbier-Chassefière et al., 2009; Ikeda et al., 2011).

Different monosaccharides have been chemically modified in order to interfere with GAG biosynthesis, chain elongation, or sulfation. One of them is peracetylated 6-fluoro-*N*-acetylgalactosamine [6F-GalNAc (Ac3)], which can potentially inhibit 6-*O* sulfation of GAG chains. As described above, the sulfation pattern is essential for interaction with various ligands as well as for pathogen adhesion. Another of these sugars, 6F-*N*-acetyl-D-galactosamine, is an inhibitor of GAG biosynthesis, which may affect the adhesion of many ligands, including bacteria (van Wijk et al., 2015).

Different antagonists of GAGs have also been designed for therapeutic purposes. Surfen, bis-2-methyl-4-amino-quinolyl-6-carbamide, is an antagonist of HS first described in 1938 as an excipient for the production of depot insulin. This molecule binds electrostatically to sulfated GAGs, competing in their interaction with different molecules, including FGF2, VEGF or glycoprotein D and, consequently, altering different processes, such as anticoagulant activity, cell adhesion to fibronectin, and viral infection by HIV-1 (Schuksz et al., 2008).

## Conclusion

The report on antimicrobial resistance made by the WHO in 2014 emphasizes a previously known problem that has increased significantly in recent years: bacterial resistance to antibiotics. PGs and GAGs present unique characteristics that allow them to play essential roles in the interaction between bacterial pathogens and host cells. The characterization of GAG-pathogen interactions has allowed the development of certain therapeutic molecules which are able to fight different infections. Further studies will lead to new therapies capable of more efficiently interfering with or blocking infections, helping to overcome antimicrobial resistance.

## Author Contributions

BG: main author of this review, conception and design, drafting the article, final approval of version to be submitted. Experience and information about bacterial attachment to eukaryotic cells through proteoglycans and glycosaminoglycans. JM-L: design, drafting the article, final approval of version to be submitted. Experience in clinical ophthalmology and about eye infections. CM: design, drafting the article, final approval of version to be submitted. Experience and information about human normal attachment to cells through glycosaminoglycans. IA: design, drafting the article, final approval of version to be submitted. Experience in clinical ophthalmology and about eye infections. LQ: design, drafting the article, final approval of version to be submitted. Expert in proteoglycans and glycosaminoglycans. FV: correspondence author, design, drafting the article, final approval of version to be submitted. Experience in clinical microbiology and infectious diseases.

## Conflict of Interest Statement

The authors declare that the research was conducted in the absence of any commercial or financial relationships that could be construed as a potential conflict of interest.
